# Time‐ and space‐resolved transcriptional regulation in *Arabidopsis thaliana*

**DOI:** 10.1111/plb.70196

**Published:** 2026-03-13

**Authors:** S. Zenker, K. Schiller, A. Bräutigam

**Affiliations:** ^1^ Department of Computational Biology, Faculty of Biology Bielefeld University Bielefeld Germany; ^2^ Department of Computational Biology, Center for Biotechnology (CeBiTec) Bielefeld University Bielefeld Germany

**Keywords:** *Arabidopsis thaliana*, diurnal, kinases, protein degradation, regulation, transcription factors

## Abstract

- General metabolism and responses to internal or external signals are tightly regulated in plants. We hypothesise that a network of intermediate regulatory proteins including transcription factors, kinases, and E3 ligases connect input signals like light, temperature, circadian clock, and ontogenetic pathways to output pathways. We therefore investigate the transcriptional dynamics of these regulators.
- RNA sequencing was performed on 3‐week‐old *Arabidopsis thaliana* Col‐0 rosettes grown under 12 h/12 h light/dark conditions sampled every 2 h over 24 h to match publicly available single‐cell data of same age plants. Both were analysed to identify the abundance of intermediate regulators in time and space.
- Intermediate regulators are of significantly lower abundance compared with other transcripts in the Arabidopsis transcriptome. More than half of expressed kinases, E3 ligases and transcriptions factors vary in either time, space or both in mature leaves.
- Dynamic expression patterns of regulators allow plants to maintain tightly regulated metabolism while providing sufficient room for specific stress responses. High plasticity of the Arabidopsis transcriptome highlights the importance of considering sampling time‐of‐day and cellular resolution for experiments.

## Graphical abstract

Transcript abundance patterns in time and space of kinases, E3 ligases and transcription factors in *Arabidopsis thaliana* demonstrate ample room for dynamic and specific regulation of metabolism and stress responses.

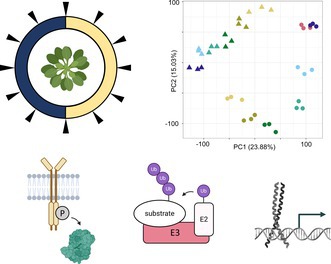

## INTRODUCTION

*Arabidopsis thaliana* contains a large proportion of genes with regulatory functions in its genome. Regulatory proteins include transcription factors (TFs), which occupy 6–7% of total genes (Riechmann *et al*. 2000; Petroll *et al*. 2025), kinases, which account for 4% (Zulawski *et al*. 2014), and the E3 ligase family which has more than 1,400 members (Vierstra 2009). Closely related TFs are often functionally redundant to each other (Leivar *et al*. 2008) by sharing binding motifs and binding sites (Zenker *et al*. 2025). Kinases phosphorylate targets comparatively unspecifically *in vitro* (Baena‐González *et al*. 2007) and can act redundantly (Tena *et al*. 2011). Expression changes in space and time have been suggested to account for specificity in the face of apparent redundancy.

Mature Arabidopsis leaves contain multiple cell types, guard cells, stomata and pavement cells in the epidermis, spongy and palisade mesophyll cells, and bundle sheath cells surrounding phloem and xylem in the veins (Lee *et al*. 2025). The cells in these tissues share features, often called housekeeping, such as DNA maintenance, protein synthesis, and others. But they also have specific features such as a cuticula maintained by epidermal cells or transport capacity needed in the phloem (Lucas *et al*. 2013; Lewandowska *et al*. 2020). Combinatorial effects of regulatory proteins are hypothesised to determine cell specificity. The differences in regulatory proteins also lead to different output to stresses in different cell types (Tenorio Berrío *et al*. 2025; Wang *et al*. 2025). In addition to this spatial diversity, cells in mature Arabidopsis leaves are governed by diurnal changes cued by light, temperature, and the circadian clock. Global transcriptomic analyses based on microarrays identify ~6–50% of expressed genes as diurnal (Harmer *et al*. 2000; Bläsing *et al*. 2005) and recent RNA‐seq based analyses 61% (Redmond *et al*. 2025). The significance of the diurnal changes in transcripts is unclear because proteomics experiments have determined that diurnal changes in the Arabidopsis leaf transcriptome and proteome do not match as most quantifiable proteins do not oscillate (Baerenfaller *et al*. 2012). The median protein half‐life is 6.3 days for quantifiable proteins (Li *et al*. 2017). The restriction ‘quantifiable’ is important in these assessments as only proteins with sufficient abundance can be reliably quantified in either approach. Proteins with regulatory functions are frequently underrepresented in these analyses (Baerenfaller *et al*. 2012; Li *et al*. 2017). We hypothesised that genes with regulatory functions are expressed at lower levels leading to reduced transcript per million contributions to the transcriptome.

Metabolism in general and responses to stresses require the regulation of proteins in time and space. Low protein degradation rates make it unlikely that protein turnover of enzymes in diurnal patterns governs the abundance of enzymes and therefore the flux (Schiller *et al*. 2025). In contrast, modulation of enzyme activity via kinases may tune activity throughout the day. For example, the enzyme phospho*enol*pyruvate carboxylase (PEPC) which produces oxaloacetate from PEP is regulated by PEPC kinase which is tightly regulated in diurnal patterns to tune day and nighttime metabolism to different photosynthetic subtypes such as C3, C4, and CAM photosynthesis (Hartwell *et al*. 1999; Aldous *et al*. 2014). For many response pathways, the ability to respond is different in time. Responses to internal (Lee *et al*. 2016), abiotic (Allen *et al*. 2006; Li *et al*. 2019) and biotic signals (Bhardwaj *et al*. 2011) are gated by the circadian clock. Protein synthesis is imbalanced between day and night (Piques *et al*. 2009; Duncan & Millar 2022), and as protein levels are fairly stable (Li *et al*. 2017), protein degradation is likely highly regulated (Vierstra 2009).

Cell identity is established during ontogeny and is, with the exception of regenerative processes, stable. For example, once mesophyll is differentiated, veins can no longer be formed (Scarpella *et al*. 2004). Prior to mesophyll differentiation, vein formation is initiated by a series of transcriptional events (Scarpella *et al*. 2004). We hypothesise that after these initial determinants during ontogeny, intermediate regulatory proteins maintain the necessary processes in mature leaves. Similarly, diurnal expression is controlled by top‐level regulation systems. The core circadian clock in Arabidopsis consists of a feedback circuit between differently phased regulators ensuring 24‐h oscillation by transcriptional control (Staiger *et al*. 2013). Components of the core clock are also regulated post‐transcriptionally, for example, Circadian Clock Associated 1 (CCA1) is phosphorylated by Casein Kinase 2 (CK2) (Daniel *et al*. 2004) and Timing of CAB Expression 1 (TOC1) can be ubiquitinylated by F‐box protein Zeitlupe (ZTL) (Harmon *et al*. 2008). Light and temperature also cue diurnal expression. Light is perceived through red and blue light receptors and its signal is transmitted to transcriptional regulators such as phytochrome interacting factor (PIF) proteins and elongated hypocotyl 5 (HY5) for red light (Leivar *et al*. 2008; Van Gelderen *et al*. 2018) and to Target of EAT1 (TOE1) for blue light receptors (Du *et al*. 2020). Temperature also feeds into PIF TFs, for example, PIF7 (Chung *et al*. 2020). Evidence for several protein families suggests that differential expression patterns in time and space are common. Many TFs downstream of the core clock show diurnal expression patterns, for example members of the C2‐like BBX‐type family often peak shortly after dawn (Balcerowicz *et al*. 2021). Downstream TFs have also been identified as locally expressed, for example, transcripts of multiple members of the NAC and R2R3 MYB family have been shown to localise in the phloem (Zhao *et al*. 2005) or the TF FAMA in guard cells (Ohashi‐Ito & Bergmann 2006).

We hypothesise that output pathways such as general metabolism and responses to signals are connected to a network of intermediate regulatory proteins including TFs, kinases, and E3 ligases, which in turn receive signals from initial top‐level regulators downstream of sensors. To contrast and compare expression patterns in space and in time, we generated a 24 h diurnal leaf transcriptomic dataset sampled every 2 h that is developmentally matched to the single‐cell leaf transcriptome of 21‐day‐old Arabidopsis rosettes (Lee *et al*. 2025). The data show that of the 770 expressed kinases, 509 vary in time, space, or both in a mature leaf. The majority peak at zt13 and 32.74% of rhythmic kinases peak between zt13 and zt15. The E3 ligases for protein degradation also vary to a large degree with 420 of 732 expressed genes varying in time, space or both. Similarly, of 1,121 expressed TFs, 708 vary in time and/or space. We conclude that at a plant age of 21 days in mature aerial tissues, a majority of regulatory transcripts vary to large degrees enabling the time‐ and space‐specific regulation of metabolism and stress response. The regulatory space not only includes transcriptional regulation, which is evident in the large proportion of the transcriptomic variation of TFs and target genes, but also post‐transcriptional regulation as evidenced by dynamically expressed low‐abundance kinases and E3 ligases.

## METHODS

### Growth conditions and sampling

*Arabidopsis thaliana* Col‐0 seeds were surface sterilized using ethanol and stratified for 2 days at 4 °C in the dark. Seeds were transferred on ½ MS Agar plates (0.8%) without addition of any sugar and grown for 3 weeks in a climate chamber with a 12 h/12 h day/night cycle: 22 °C, 120 μE and 18 °C, 0 μE. Relative humidity was constant at 50%. Above‐ground material from three whole plates was harvested every 2 h as three biological replicates by flooding the plate with liquid nitrogen. Sampling was started at Zeitgeber time 2 (zt2) and ended with a repetition of zt2* the next day. Zt12 was sampled shortly before darkness and zt0 directly before the lights turned on. Transitions were immediate with no light gradient applied. RNA was extracted using Qiagen RNeasy Plant Mini Kit and a 15 min on‐column DNaseI digestion. RNA library was prepared using Illumina XLEAP chemistry and sequenced as single strands on a NextSeq2000 yielding 6.5 to 12.7 million reads per sample.

### Data analysis

Raw reads were aligned to the TAIR10 Arabidopsis transcriptome of only primary transcripts with kallisto v0.44.0 (Bray *et al*. 2016) with the parameters ‐‐single ‐l 200 ‐s 20 resulting in mapping rates of 92.4–94.8%. Further downstream analyses were performed using R v4.5.1 (R Core Team 2024) and the tidyverse (Wickham *et al*. 2019). A PCA was computed with prcomp on scaled and centred expression data. To determine rhythmic transcripts, JTK_CYCLE (Hughes *et al*. 2010) was used on expressed transcripts, here defined as >1 tpm in all three replicates of at least one time point. A target period length of 24 h was set and transcripts with Benjamini–Hochberg adjusted *P*‐value < 0.01 and an amplitude >1 were considered diurnal. For assessment of robustness, two shifted sets of 4 h intervals were subsampled and also tested with JTK_CYCLE. Transcriptomic Zeitgeber time was predicted using the ensemble of 100 pretrained RNA‐seq models from ChronoGauge (Reynolds *et al*. 2025). GO term enrichments were performed using topGO (Adrian Alexa 2017) on all genes peaking at a given Zeitgeber time and corrected for multiple hypothesis testing using Benjamini–Hochberg correction, subsequently defining a q‐value < 0.05 as significantly enriched. To analyse spatial resolution, data from Lee *et al*. (2025) was reanalysed. The Seurat object of single‐cell RNA‐seq data of 21‐day‐old rosettes was accessed from GSE226097 and cell types were assigned based on Lee *et al*. (2025). Cell type‐specific expressed genes were identified using FindAllMarkers from the Seurat v5.3.0 R package (Hao *et al*. 2024) with min.pct = 0.1, logfc.threshold = 0.25 and onl.pos = T. Genes with a *P*‐value < 0.05 in not more than two cell types were defined as spatially specific. For the fraction of cells with expression, a gene was considered expressed if at least one transcript was detected in a cell.

To annotate the intermediate regulators, TFs in *A. thaliana* were retrieved from the TAPscan v4 database (Petroll *et al*. 2025). Proteins with kinase domains were selected via HMMER search using the HMM models for Pfam domains Pkinase (PF00069) and PK_Tyr_Ser‐Thr (PF07714) and default settings (Finn *et al*. 2011). Subclade annotations of kinases were retrieved from Zulawski *et al*. (2014). E3 ubiquitin ligases mediating substrate specificity, namely, HECT, RING, F‐box, U‐box, BTB, DDB1 proteins and APC complex recognition subunits (Vierstra 2009) were retrieved from Mazzucotelli *et al*. (2006). Joined heatmaps of diurnal and spatial data were generated with the R package ComplexHeatmap (Gu 2022) from z‐scores of diurnal genes with Euclidean distance and Ward's Clustering. All processed data are available in Table S1.

## RESULTS

**Fig. 1:**
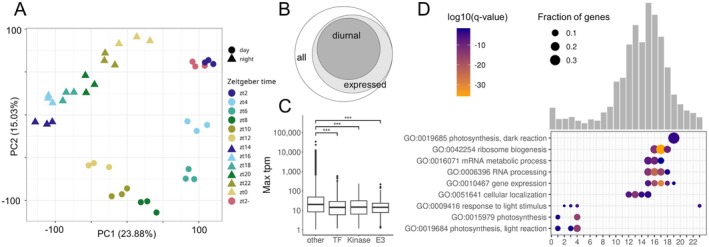
Global diurnal expression patterns in the Arabidopsis transcriptome. (A) PCA of 13 sampled time points with three biological replicates each. Coloured by Zeitgeber time with shape indicating day and night. (B) Euler diagram showing the proportions of expressed and diurnal transcripts in Arabidopsis leaves. (C) Boxplots of maximal expression levels per gene per day. Wilcoxon test compares kinases, E3 ligases and TFs to all other genes (***P < 0.001). (D) Number of diurnal transcripts grouped by their peak phase as defined by JTK_CYCLE (Table S2) indicated by bars. Selected enriched GO terms (q‐value < 0.05) per phase are shown with size indicating fraction of phased genes in GO term and colour encoding log10 q‐value.

To test the diurnal dataset for overall expression patterns, a principal component analysis (PCA) and phasing of transcripts were analysed. The PCA partitioned 38.91% of the variation in the first two components (Fig. 1A). Transcriptomic changes are stable across biological replicates and follow a 24 h‐period reaching zt2 again at zt2* the following day. The pattern resembles a circle in the first two principal components (Fig. 1A). Samples taken in the dark are clearly separated from those in the light (Fig. 1A). Out of 27,206 nuclear protein coding genes, 18,337 (67.4%) genes are detectable at >1 tpm at least at one time point in aerial parts of 21‐day old Arabidopsis plants (Table S1), of which 62% (11,364) are detected as diurnal (q‐value < 0.01; amplitude >1) within a 24‐h period by JTK_CYCLE (Fig. 1B, Table S2). The majority of diurnal transcripts have an amplitude between one and 10 (Fig. S1A). Both subsampled 4 h intervals result in fewer diurnal transcripts (7,861 and 7,657), of which 96.7% and 94.3% are shared with the 2 h interval set (Fig. S1B). Circadian time predicted by ChronoGauge (Reynolds *et al*. 2025) deviates by a mean error of −5.7 min across all time points and replicates from the sampling time (Fig. S1C, Table S3). Predicted Zeitgeber times of zt18 and zt22 have the highest error of around 100 min, while all others are below 60 min (Fig. S1C, Table S3). Regulatory transcripts are of significantly lower abundance compared with all transcripts (*P* = 1.01e‐26 for TFs, *P* = 1.34e‐15 for kinases, and *P* = 2.53e‐32 for E3 ligases, Fig. 1C). Despite mean abundance differences of 60.74, 63.03 and 67.61 tpm between TFs, kinases and E3 ligases compared with the whole transcriptome respectively, the effect sizes are small (Cohen's d = −0.12 for TFs, d = −0.12 for kinases, d = −0.13 for E3 ligases). A GC content bias for transcript abundance could not be detected (Pearson correlation r = 0.07) but transcripts of E3 ligases have significantly higher GC content compared with all transcripts (Fig. S1D, *P*‐value < 0.05). The largest number of diurnal transcripts peak at the beginning and middle of the night (Fig. 1D). Consequently, genes with peak expression at zt15, zt16, and zt17 enrich in GO terms related to mRNA metabolic processes (Fig. 1D, Table S4). The GO term photosynthesis is enriched at zt1‐zt4 while response to light stimulus is already enriched 1 h before lights turned on at zt23 (Fig. 1D, Table S4). GO terms associated with the carbon reactions enrich during the dark period at zt19 (Fig. 1D, Table S4).

### Kinases in time and space

**Fig. 2:**
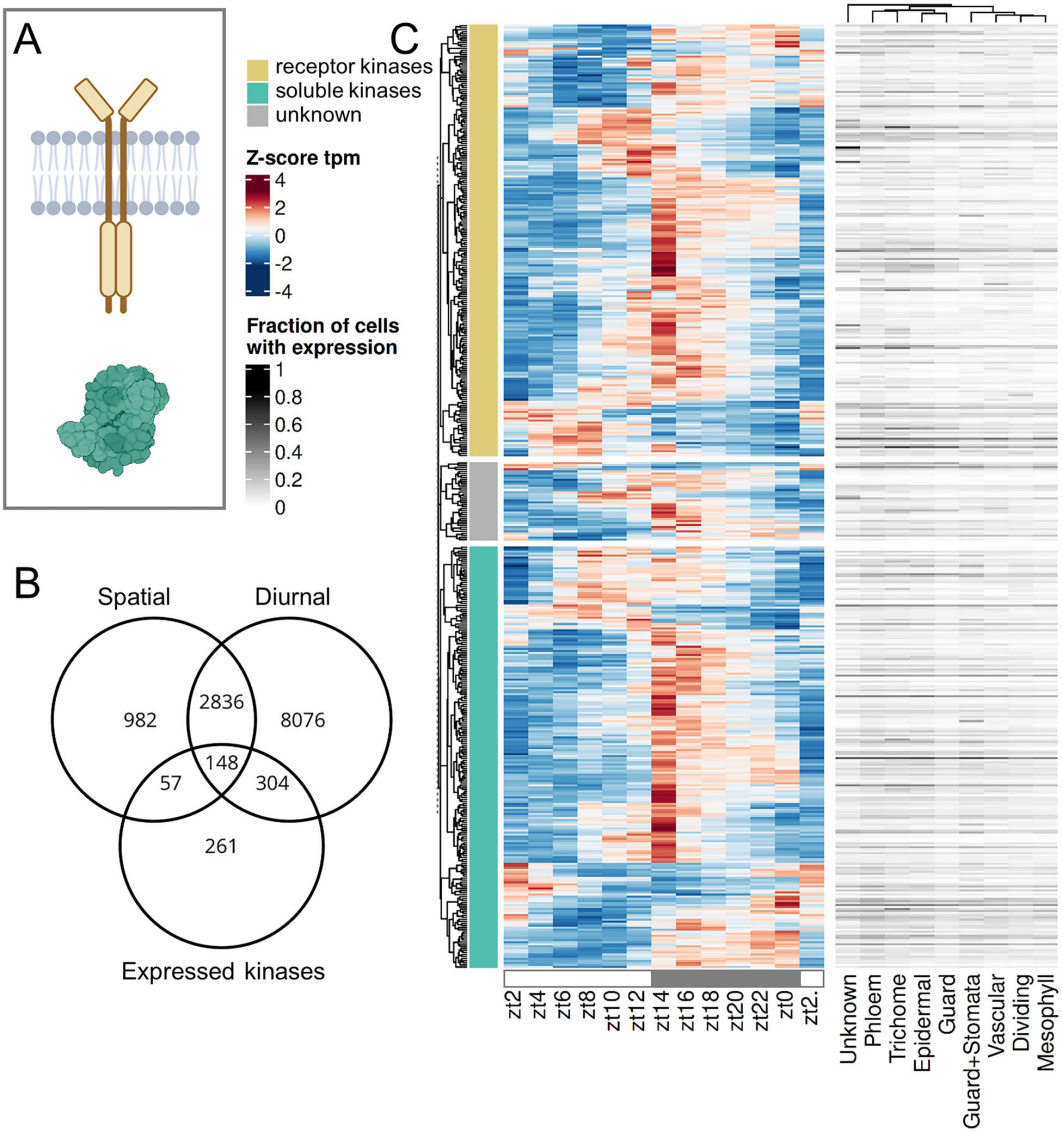
Expression patterns of kinases. (A) Schematic visualization of a membrane‐located and a soluble kinase as the two main subtypes (Zulawski *et al*. 2014). (B) Venn diagram showing the overlap between diurnal, spatial cell type‐specific genes, and expressed kinases. (C) Heatmap showing expression (z‐score tpm) of all diurnal kinase transcripts and fraction of cells with expression for each cell type (Lee *et al*. 2025) grouped by subclades (Zulawski *et al*. 2014).

To test kinases for their variation in time and space (Fig. 2), they were grouped into 559 receptor kinases and 381 soluble kinases based on Zulawski *et al*. (2014). We find 770 of 1,051 kinases expressed and 452 of those (58.7%) to be diurnal (Fig. 2B). Subsets of 209 receptor kinases (37.39%) and 205 soluble kinases (53.8%) are diurnal (q‐value < 0.01; amplitude >1). The heatmap of diurnal transcript levels (Fig. 2C) shows that different kinases peak in abundance during different times of the day, but the majority of 35.84% (162) peak around the beginning of the night (zt13‐zt15). This is true for both subclades of kinases, receptor kinases and soluble kinases (Fig. 2C). Only 8.8% of the diurnal soluble kinases peak in the first 8 h of the day but 21.95% peak in the final 4 h of the day. Of the diurnal receptor kinases, 3.45% peak in the first 4 h of the day, 8.13% peak around noon, and 25.36% peak in the final 4 h of the day. Soluble kinases and receptor kinases which peak at the beginning of the night frequently remain elevated in abundance throughout the night compared with their daytime abundance (Fig. 2C). A small group of kinases peak during the end of the night and continue into the day (Fig. 2C). Among the soluble kinases, ABA‐activated kinase Open Stomata 1 (OST1; AT4G33950) which phosphorylates Slow Anion Channel‐Associated 1 (SLAC1) peaks at zt17 and is expressed ubiquitously in all cell types (Fig. S2). Mitogen‐activated Protein Kinase 3 (MPK3; AT3G45640) and MPK6 (AT2G43790) involved in pathogen‐associated molecular pattern recognition peak at zt10 and zt15 respectively (Fig. S2). MPK3 is significantly expressed in trichome cells but MPK6 is not cell type‐specific (Fig. S2). PEPC kinase (AT1G08650) peaks at zt7 and is not cell type‐specific (Fig. S2). The receptor kinase Brassinosteroid Insensitive 1 (BRI1; AT4G39400) involved in brassinosteroid signalling peaks at zt13 and is cell type‐specific in the phloem and trichome (Fig. S2). Receptor kinase Flagellin‐sensitive 2 (FLS2; AT5G46330) peaks at zt13 and is significantly differentially expressed in the phloem (36% of cells), but also found in 19–26% of cells of other types (Fig. S2). The phloem overall has the highest amount of spatially specific genes with 2,701 genes, followed by 563 in the trichome (Table S5). Similar to these single gene examples, the majority of leaf kinases are dynamic in time, space, or in both. If spatial specificity is defined as differentially expressed in a maximum two cell types, 205 expressed kinases are spatially specific (q‐value < 0.01; Table S5). A subset of 148 of the 452 diurnal kinases are diurnal and spatial (Fig. 2B). In summary, 66.1% of the kinases expressed in 21‐day old aerial parts of Arabidopsis are dynamic in time, space or both. Remarkably, on/off behaviour was detected in 74 of the 770 expressed kinases with an average expression of <1 tpm at least one time point during the diurnal time‐course.

### E3 ligases in time and space

**Fig. 3:**
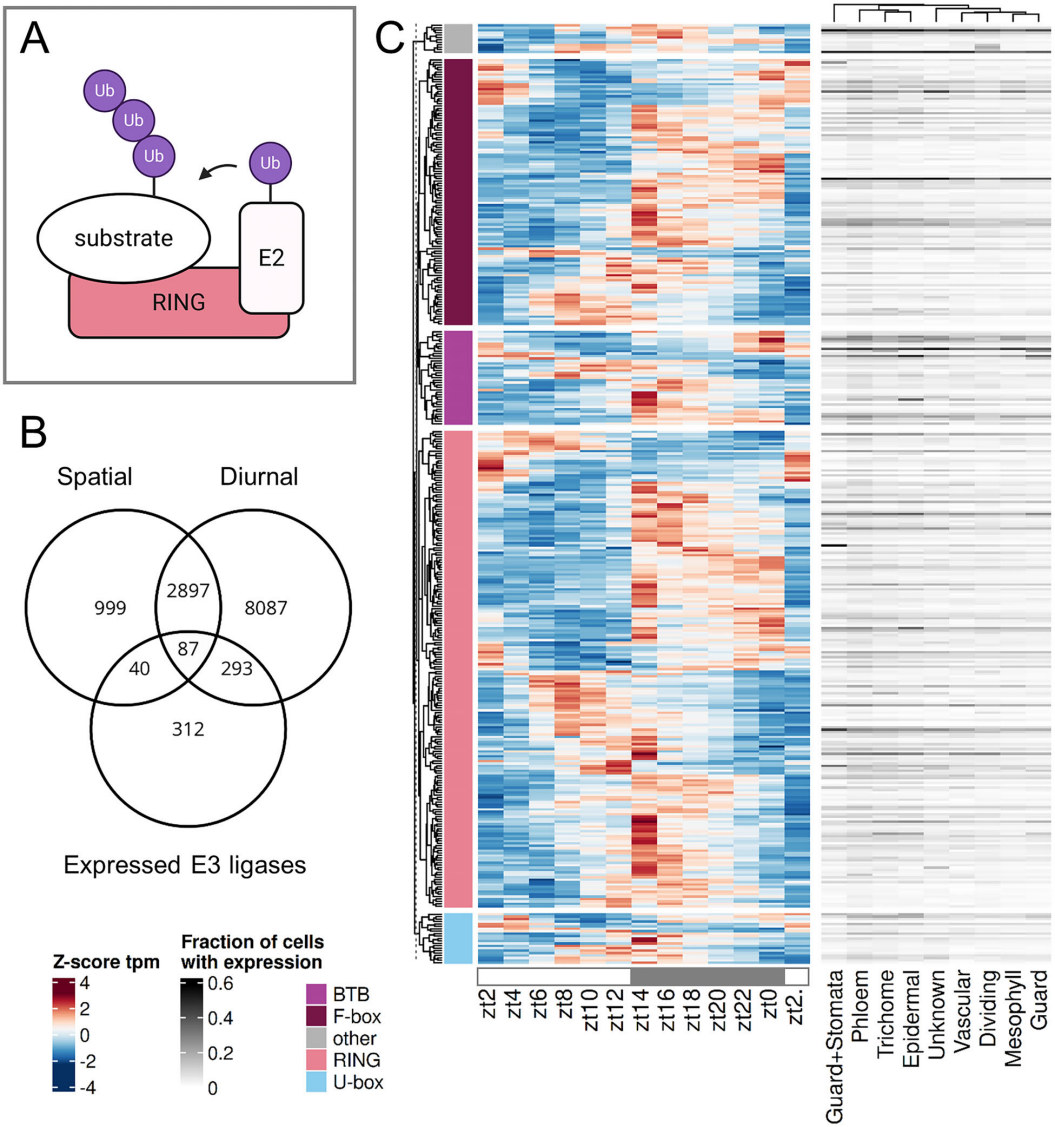
Expression patterns of E3 ligases. (A) Schematic visualization of the substrate specific ubiquitinylation by a RING‐type ligase. (B) Venn diagram showing the overlap between diurnal, spatial cell type‐specific genes, and expressed E3 ligases. (C) Heatmap of all diurnal transcripts (z‐score tpm) over time and fraction of cells with expression for each cell type (Lee *et al*. 2025). Annotations of the four largest subgroups are colour‐coded and proteins of smaller subgroups are combined under other.

Single protein E3 ligases (*e.g*. RING; Fig. 3A) and the target specificity mediating proteins from E3 ligase complexes (hereafter E3 ligases) were tested for their variation in time and space (Fig. 3). Seven HECT, 486 RING, 49 U‐box, 81 BTB, 5 DDB1, 700 F‐box and 10 other nuclear protein coding genes were annotated based on Mazzucotelli *et al*. (2006). In total, we find 732 of 1,338 E3 ligases expressed in 21‐day‐old rosettes and 380 (51.91%) to be diurnal (q‐value < 0.01; amplitude >1, Fig. 3B). Different E3 ligases peak in transcript abundance during different times of the day (Fig. 3C), but the majority of 28.35% peak around the beginning of the night (zt13‐zt15). A small subset of 3.41% of the diurnal E3 ligases peak in the first 4 h of the day, 4.46% peak around noon, and 21.8% peak in the final 4 h of the day. Transcript levels of F‐box, RING and BTB proteins which peak at the beginning of the night frequently remain elevated in abundance throughout the night, which is less observed for U‐box proteins (Fig. 3C). Specifically, within the largest subgroup of F‐box proteins, 2.67% of the diurnal transcripts peak in the first 4 h of the day, as well as at noon, and 18.75% peak in the final 4 h of the day. The auxin receptor F‐box protein Transport Inhibitor Response 1 (TIR1; AT3G62980) peaks at zt17 and is cell type‐specific in phloem and epidermal cells in 21‐day‐old rosettes (Fig. S2). The jasmonate receptor Coronatine Insensitive 1 (COI1; AT2G39940) is not diurnal but is cell type‐specific in the phloem (Fig. S2). The E3 ligase Constitutive Photomorphogenic 1 (COP1; AT2G32950) is also not diurnal but cell type‐specific in the phloem and trichome (Fig. S2). Transcripts of High Expression of Osmotically Responsive Genes 1 (HOS1; AT2G39810), which is a negative regulator of cold tolerance, peak at zt16 and are detected in ~60% of guard and stomatal cells and significantly enrich for this cell type (Fig. S2, Table S1). We find 17.35% of transcripts of E3 ligase genes spatially specific and a subset of 87 of the 732 diurnal transcripts are not only diurnal but also spatially specific (Fig. 3B). In summary, 57.38% of the transcripts for proteins mediating the specificity in protein degradation expressed in 21‐day old aerial parts of Arabidopsis are dynamic in time or space or both. An on/off behaviour was detected in 66 of the 732 expressed E3 ligases.

### Transcription factors in time and space

**Fig. 4:**
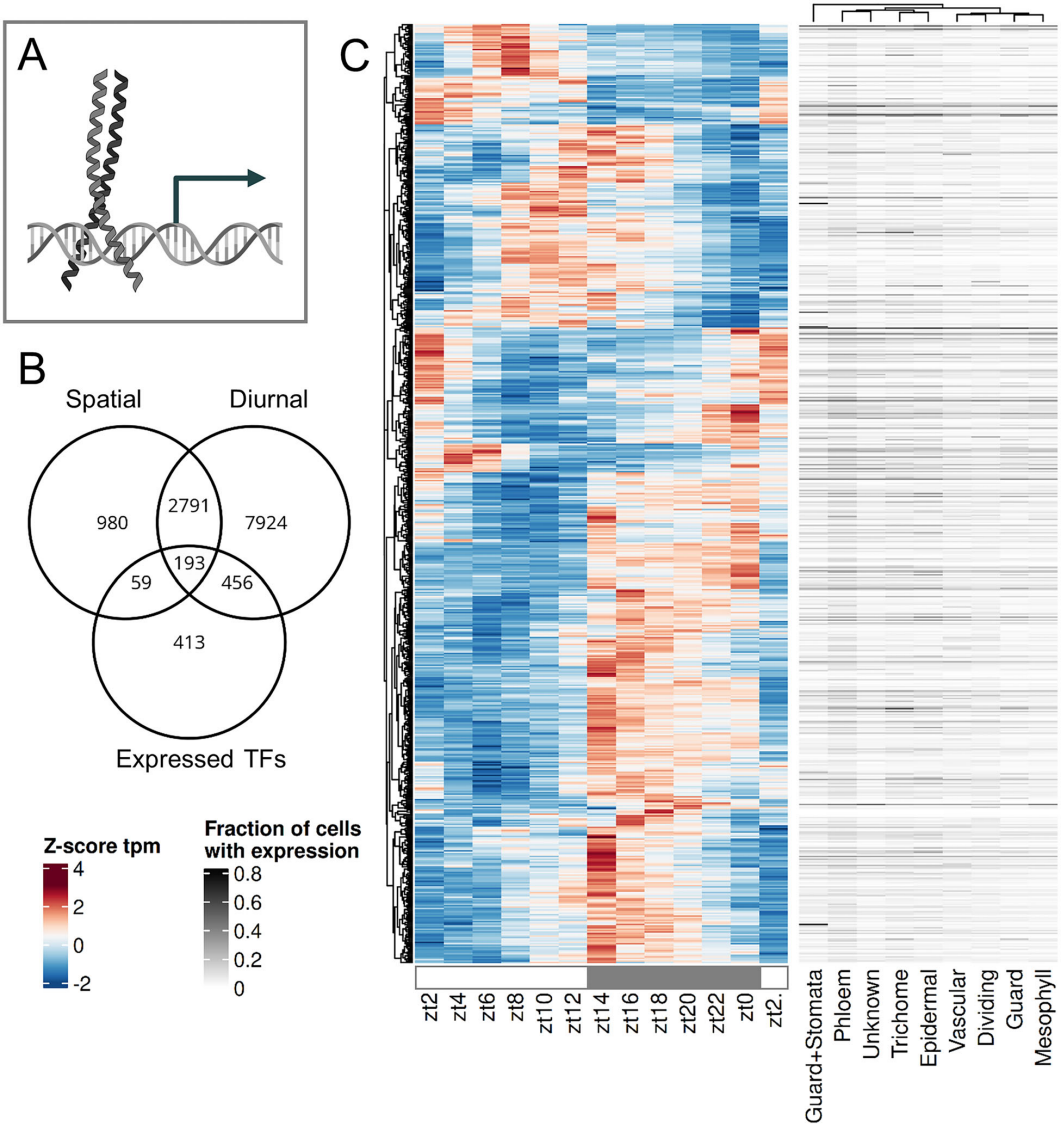
Expression patterns of transcription factors. (A) Schematic visualization of a DNA‐binding TF, in this case a bZIP‐type. (B) Venn diagram showing the overlap between diurnal, spatial cell type‐specific transcripts, and expressed TFs. (C) Heatmap of expression (z‐score tpm) of all diurnal transcripts over time and fraction of cells with expression for each cell type (Lee *et al*. 2025).

Transcription factors (TFs) were tested for their variation in time and space (Fig. 4). Only DNA‐binding TFs were considered (Fig. 4A). We find 1,121 of 1,725 DNA‐binding TFs expressed in aerial tissues and 649 (57.89%) to be diurnal (Fig. 4B). The heatmap shows that transcript abundance of different TFs peak during different times of the day (Fig. 3C). 6.47% of the diurnal TFs peak in the first 4 h of the day, 6.16% peak around noon, and 20.19% peak in the final 4 h of the day while 32.67% peak in the first 4 h of the night, 23.42% at midnight, and 11.09% at the end of the night (Fig. 4C). A small group of TFs peak towards the end of the night and into the day (Fig. 4C). The top‐level circadian clock regulators peak at zt22 (LHY), zt23 (CCA1), and at zt12 (TOC1) and are generally expressed in all cell types (Fig. S3, Table S1), but LHY and CCA1 are also differentially expressed in the phloem (Fig. S2). Peak phases of 13 circadian clock genes are within ±3 h of previously defined expected peak phases under 12 h/12 h light/dark conditions (Table S6), despite variation in temperature entrainment (here 22 °C/18 °C *versus* 22 °C/12 °C Michael *et al*. 2008).

The TF FAMA (AT3G24140) is a known regulator of guard cell fate which peaks at zt14 and is generally more expressed during the night (Fig. S2). Transcripts significantly enrich in the guard and stomata lineage cluster, where at least one transcript is detected in 83% of cells (Fig. S2, Table S1). The photosynthetic transcription factor Golden2‐like 1 (GLK1; AT2G20570) peaks at zt6 and is enriched in the phloem (Fig. S2). Similar to these TFs, the majority of transcripts for TFs are dynamic in time, in space, or in both. If spatially specific is defined as in maximally two cell types, 22.48% of TFs are spatial (Fig. 4B). A subset of 193 TF transcripts are both diurnal and spatial (Fig. 4B). In summary, 63.16% of the transcripts for TFs expressed in 21‐day‐old aerial parts of Arabidopsis are dynamic in time or space or both. Transcripts for TFs show on/off behaviour with 187 of the 1,121 expressed ones with an average expression of <1 tpm at least one time point during the diurnal time‐course.

## DISCUSSION

The data for diurnal expression patterns of the Arabidopsis rosettes over 24 h grown under 12 h light/12 h dark growth conditions (Table S1) combined with the matched single‐cell atlas (Lee *et al*. 2025) enable the analysis of the intermediate regulatory layer (Figs 2, 3, 4). The analysis detects 62% of transcripts as diurnal (Fig. 1B, Table S2) which is comparable to Redmond *et al*. (2025) and broadly comparable to microarray datasets (Harmer *et al*. 2000; Bläsing *et al*. 2005). Expectedly, lower sampling resolution of 4 h intervals result in fewer diurnal transcripts, but almost full subsets of the 2 h sampling (Fig. S1B), suggesting robust rhythmic patterning. Robust diurnal expression is also supported by overall small deviation of predicted from sampled Zeitgeber times (Fig. S1D, Table S3), as well as matching peak phases of known clock transcripts (Fig. S3, Table S6). If cut‐off values for diurnal rhythmicity are relaxed (see Methods Section) an even larger proportion of the transcriptome would be considered diurnal. This proportion massively exceeds the known direct targets of the circadian clock (Nagel *et al*. 2015; Ezer *et al*. 2017). This observation is likely explained by the large proportion of diurnal TFs (Fig. 4). Given that many TFs share target binding motifs and sites (Zenker *et al*. 2025) a large proportion of TFs with diurnal behaviour will result in a large proportion of the total transcriptome with some degree of rhythmic expression (Fig. 1B).

PEPC kinase is one example for a regulatory kinase which directs metabolic flow (Schiller & Bräutigam 2021). The kinase has on/off behaviour in CAM plants being on during the night (Hartwell *et al*. 1999). In stark contrast, transcripts peak during the day in the C3 plant Arabidopsis at zt7 but are not on/off (Fig. S2, Table S1), which is characteristic for a C3 plant (Fontaine *et al*. 2002) and highlights the different needs of regulation for PEPC in a C3 background. Seventy‐four kinases show on/off behaviour in Arabidopsis aerial tissue during a day/night time‐course similar to the PEPC kinase in CAM plants and may perform similar roles for different processes and fluxes. FLS2, the pathogen‐associated pattern recognition sensor which recognized flagellin, surprisingly peaks during the early night but does not show on/off behaviour (Fig. S2, Table S1). Given that stomata close during the night, this expression likely does not relate to function. This behaviour highlights that a proportion of diurnal changes may not be related to function of the protein itself but is a function of the large proportion of TFs with diurnal behaviour (Fig. 4) and their partial promiscuity (Zenker *et al*. 2025). OST1 extended roles beyond stomatal regulation (Mustilli *et al*. 2002) is highlighted by its expression throughout the leaf cell types (Fig. S2, Table S1). This suggests that some but not all observed patterns for kinase transcript abundances (Fig. 2) have functional significance.

Timed experiments with protein synthesis have suggested that protein degradation is regulated diurnally (Piques *et al*. 2009; Ishihara *et al*. 2015). Indeed, there is very large potential for targeted degradation as 1,338 target specificity mediating proteins from E3 ligase complexes are annotated (Mazzucotelli *et al*. 2006; Vierstra 2009). Of these, 380 have diurnal behaviour but the majority of them peaks at the beginning of the night (Fig. 3C). To balance protein synthesis as suggested (Ishihara *et al*. 2015), a peak of targeted degradation during the day is required. The balancing function is thus carried out by a minority of E3 ligases (Fig. 3C). The majority peak of E3 ligases at the beginning of the night coincides with the majority peak of kinases at the beginning of the night (Figs 2C and 3C) which may suggest that the kinase based signalling may be subject to rapid and targeted turnover by E3 ligases. The fact that all signalling proteins tested have significantly lower transcript abundance (Fig. 1C) combined with the observation that protein abundance positively correlates with transcript abundance (Mergner *et al*. 2020) makes signalling proteins subject to a potentially rapid rate of turnover undetected by proteomics experiments as these quantify mostly abundant proteins.

Using this time‐ and space‐resolved data, it is possible to confirm known spatiotemporal expression patterns but also define a distinct pattern or absence thereof for other genes. For example, FAMA is a known determinant of guard cell fate and generally used to annotate the guard and stomata lineage clusters in single‐cell analyses (Ohashi‐Ito & Bergmann 2006; Lee *et al*. 2025). Here, we show that FAMA expression peaks during the night (Fig. S2, Table S2). HOS1 transcripts peak at zt16 (Fig. S2, Table S2) which might be a gating mechanism of Arabidopsis. *Hos1* mutant plants have reduced freezing tolerance (Ishitani *et al*. 1998) which could in turn indicate that by peak expression in the night the plant reacts to lower temperatures which are usually perceived during the night. Temperature, light, and core clock expression change at dusk and dawn, leading to the expectation of two time points of diurnal transcript peaks in downstream targets. Previous analyses with microarrays identify a strong peak in the morning for all diurnal transcripts under the same photoperiod (Bläsing *et al*. 2005). The RNA‐seq data acquired here presents its peak in diurnal transcripts at the beginning and during the night (Fig. 1D). Both the growth conditions which differ in this experiment compared with Bläsing *et al*. (2005) in lighting by LEDs *versus* fluorescent light, entrainment (22 °C/18 °C) *versus* constant temperature (20 °C), soil *versus* plates, 50% *versus* 60–70% relative humidity, age and the technical differences with RNA‐seq no longer biasing against very low‐abundance and very high‐abundance transcripts may have contributed to the observed differences. The peak‐at‐night pattern is especially present in kinases with strong peaks in transcript abundance at zt14 (Fig. 2C), but also in E3 ligases and TFs. Kinases expressed in mature leaves also show the most spatially resolved genes (26.6%) compared with TFs (22.48%) and lastly E3 ligases with only 17.35%, indicating importance of phosphorylation for nuanced regulation in Arabidopsis.

The temporal expression pattern of core clock components has been analysed in detail (reviewed in Staiger *et al*. 2013), while the spatial expression pattern has gained less attention. We show here that the core clock components like CCA1, LHY, or TOC1 are highly temporal (Fig. S3, Table S6) with expected peak phases (Michael *et al*. 2008; Staiger *et al*. 2013), but ubiquitously expressed in all cell types (Fig. S2). This underlines that every cell expresses the components to keep the time and matches previous analyses demonstrating cell‐type‐specific circadian rhythms (Endo *et al*. 2014; Qin *et al*. 2025). Clock TFs LHY, CCA1, PRR7, and PRR9 are significantly differential in the phloem (Fig. S2, Table S5), which could be linked to a dominant vasculature clock identified previously (Endo *et al*. 2014). The phloem generally has the most differentially expressed genes (Table S5). This may reflect an only partially recognized role of the phloem as a signalling hub or it may point to technical challenges with spatial transcriptomics. Spatial transcriptomics data are often skewed towards annotating higher abundance transcripts as differential and dependent on high quality cell type annotation to reduce noise (Lähnemann *et al*. 2020). To avoid bias towards high abundance transcripts, we added the fraction of cells with expression as an alternative measure (Table S1), which considers only on/off states for each gene per cell. This measure also captures known enrichments of HOS1 and FAMA in the guard cells but shows a more ubiquitous expression of many spatially differential transcripts including core clock regulators (Fig. S2).

The proportion of spatially expressed genes is generally lower compared with diurnal numbers (Figs 2B, 3B and 4B). This could be a result of general underrepresentation of low abundance transcripts in single‐cell data (Lähnemann *et al*. 2020) and the transcripts of the regulatory genes examined here being of significantly low abundance (Fig. 1C) further reducing statistical power. As many transcripts also oscillate on a cell type level (Endo *et al*. 2014; Qin *et al*. 2025), sampling time of the single‐cell samples additionally limits transcript detection and needs to be taken into consideration for bulk and single‐cell experiments. For the single‐cell data reanalysed here (Lee *et al*. 2025), no time of sampling relative to the 16 h photoperiod was provided. Given that CCA1 and PRR7 are expressed in many cells of all cell types, and PRR9 as well to a lesser degree, but ELF3 barely and ELF4 completely absent (Fig. S2), this best matches the observed clock transcript state shortly after lights turned on at zt2 in our time‐course (Fig. S3). Although long day time‐course analyses result in overall similar diurnal transcript numbers (Redmond *et al*. 2025), length of day and temperature influence clock entrainment resulting in observable expression differences (Michael *et al*. 2008; Oravec & Greenham 2022).

We show that overall, transcripts of proteins mediating the intermediate layer of regulation are of lower transcript abundance compared with all other genes (Fig. 1C). Using bulk RNA‐seq with sufficient read depth and 2 h sampling resolution, the data identifies diurnal rhythmicity in all intermediate regulatory sets for 52% (E3 ligases), 58% (kinases) and 59% (TFs) of expressed genes, which is slightly below the 62% of all expressed genes. Combining both diurnal and spatial datasets, we are able to resolve 66.1% of kinases (Fig. 2B), 57.3% of E3 ligases (Fig. 3B) and 63.2% of TFs (Fig. 4B) in time, space, or both. This dynamically expressed intermediate regulatory layer provides ample room to maintain tight regulation of general metabolism, development, and stress response via transcription, phosphorylation, and protein degradation. The time‐course integrated with spatial data serves as an accessible resource (Table S1) to the community to identify rhythmic expression pattern in gene sets of choice.

## AUTHOR CONTRIBUTIONS

**SZ**: Data curation; formal analysis (lead); investigation; visualization; writing— original draft, review and editing. **KS**: Data curation; formal analysis (supporting); investigation; visualization; writing—original draft, review and editing. **AB**: Conceptualization; funding acquisition; project administration; resources; supervision; writing—original draft, review and editing.

## FUNDING INFORMATION

SZ is funded by the German Research Foundation (Deutsche Forschungsgemeinschaft; DFG) via grant TRR175‐D04 (INST 86/2288‐1 to AB): ‘The Green Hub, Central Coordinator of Acclimation in Plants’.

## CONFLICT OF INTEREST STATEMENT

The authors declare no competing interests.

## Supporting information


**Fig. S1.** Robustness assessment of rhythmic transcript definition. (A) Amplitude distribution of all expressed transcripts. The y‐axis is log‐scaled. (B) GC content in regulatory sets compared with the whole transcriptome tested for significance using Wilcoxon test. (C) Venn diagram showing diurnal transcripts (JTK_CYCLE q‐value < 0.01 and amplitude > 1) of shifted 4 h intervals compared with the complete set of 2 h intervals. (D) Prediction accuracy of ChronoGauge's pretrained RNA‐seq models tested on the time‐course visualized by error in minutes summarized as a boxplot and as points for each replicate at a given timepoint.

**Fig. S2.** Heatmap of commonly known examples of kinases, E3 ligases and transcription factors. Heatmap showing expression (z‐score tpm) of all diurnal transcripts and fraction of cells with expression for each cell type. Stars indicate significantly differential expression in the cell type. Single‐cell data reanalysed from (Lee *et al*. 2025).

**Fig. S3.** Expression patterns of core clock regulators. Transcripts per million (tpm) of all three replicates at each Zeitgeber timepoint. Mean values per timepoint are connected by a line.

**Table S1.** Transcripts per million over time and expressed cell ratio for all genes. Tpm values for all Arabidopsis loci mapped with kallisto on primary transcripts. Timepoint is indicated by zt (Zeitgeber time; hours after lights turned on) and triplicates are denoted by R1/2/3. Expressed cell ratio was calculated from single‐cell data of 21‐day‐old Arabidopsis rosettes reanalysed from Lee *et al*. (2025) A single‐cell, spatial transcriptomic atlas of the Arabidopsis life cycle. Nature Plants. https://doi.org/10.1038/s41477‐025‐02072‐z.
**Table S2.** Analysis of rhythmic genes from JTKcycle. Output from JTK_CYCLE analysis on expressed genes (tpm ≥ 1 in all three replicates for at least one timepoint).
**Table S3.** Predicted transcriptomic Zeitgeber times. Summary of pretrained RNA‐seq model ensemble from ChronoGauge (Reynolds *et al*. 2025 doi:10.1038/s41467‐025‐62196‐w). CT: circadian time.
**Table S4.** Enriched GO terms over time. GO term enrichments on genes with peak at given phase (JTK_CYCLE output, Table S2) calculated with topGO and filtered for q‐value<0.05 (Benjamini–Hochberg corrected).
**Table S5.** Differentially expressed genes in cell types. Cell type‐specific genes as defined by FindAllMarkers output from Seurat, filtered for markers in maximally two cell types and *P*_val_adj <0.05. Seurat object of snRNA‐seq data of 21‐day‐old rosettes from GSE226097 and cell type annotations from Table S2 were retrieved from Lee *et al*. (2025).
**Table S6.** Comparison of peak phases of known circadian clock regulators. Zeitgeber times of peak phases identified in this study compared with a microarray dataset entrained under 12 h/12 h light/dark 22 °C/12 °C from Michael *et al*. (2008) doi: 10.1371/journal.pgen.0040014.

## Data Availability

Raw reads are available under ARC (https://git.nfdi4plants.org/sanja.zenker/ath_timecourse) and NCBI SRA Bioproject PRJNA1333938. For easy access of the time‐course data, we provide two shiny applications for local execution within the ARC: one to visualize tpms of individual Arabidopsis gene identifiers over time and another one to generate a heatmap for individual gene sets. Both applications with processed data and the exemplary gene sets used in this study can be downloaded from the ARC (https://git.nfdi4plants.org/sanja.zenker/ath_timecourse).
