# Supplementary figures and images for: Time‐ and space‐resolved transcriptional regulation in Arabidopsis thaliana
Source: Plant Biol (Stuttg). 2026 Mar 13;28(4):1005–15. doi: 10.1111/plb.70196 (PMC13175949; doi:10.1111/plb.70196)

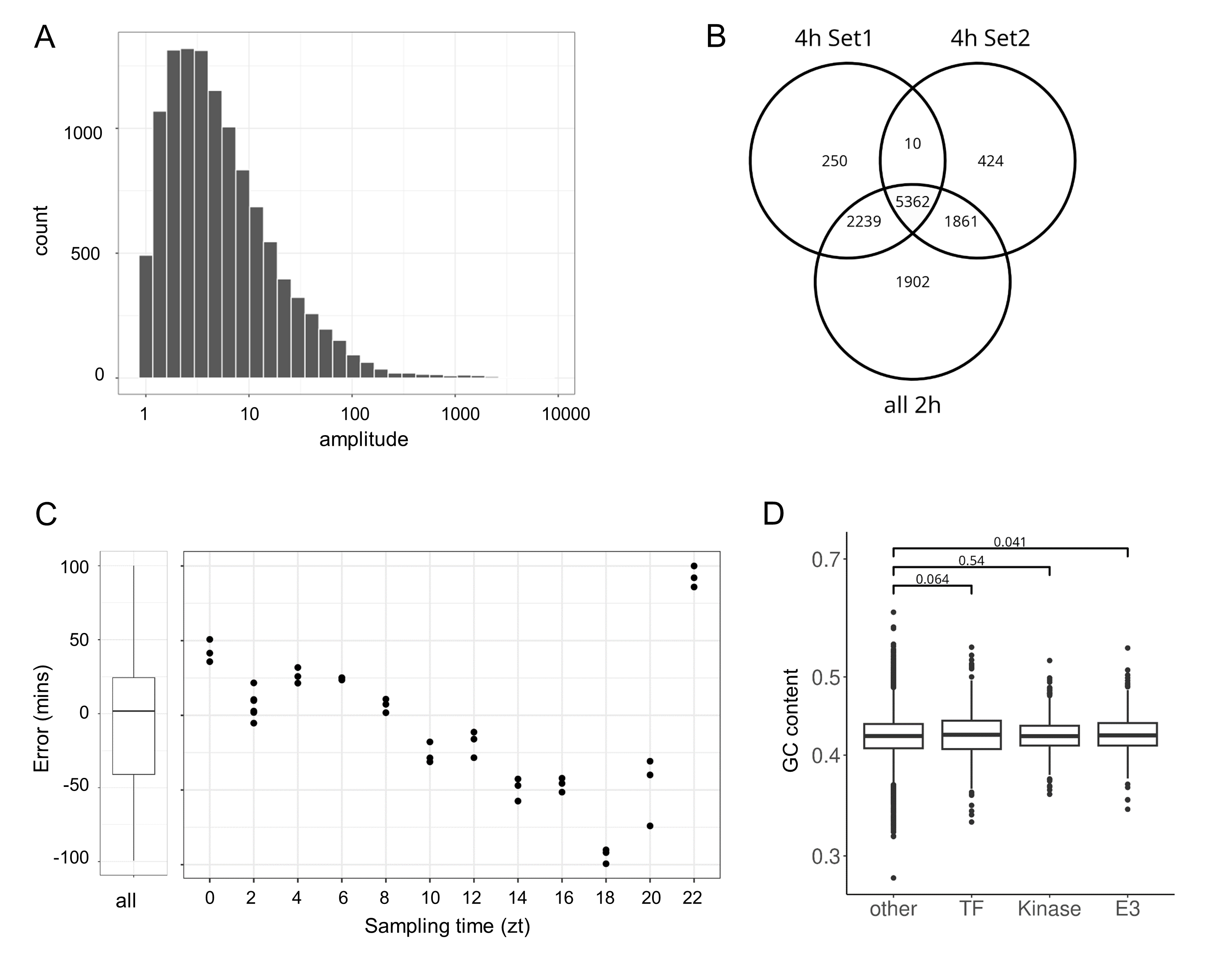

Supplement: Supplementary file 1 — Fig. S1. Robustness assessment of rhythmic transcript definition. (A) Amplitude distribution of all expressed transcripts. The y‐axis is log‐scaled. (B) GC content in regulatory sets compared with the whole transcriptome tested for significance using Wilcoxon test. (C) Venn diagram showing diurnal transcripts (JTK_CYCLE q‐value < 0.01 and amplitude > 1) of shifted 4 h intervals compared with the complete set of 2 h intervals. (D) Prediction accuracy of ChronoGauge's pretrained RNA‐seq models tested on the time‐course visualized by error in minutes summarized as a boxplot and as points for each replicate at a given timepoint. [file PLB-28-1005-s004.tif]

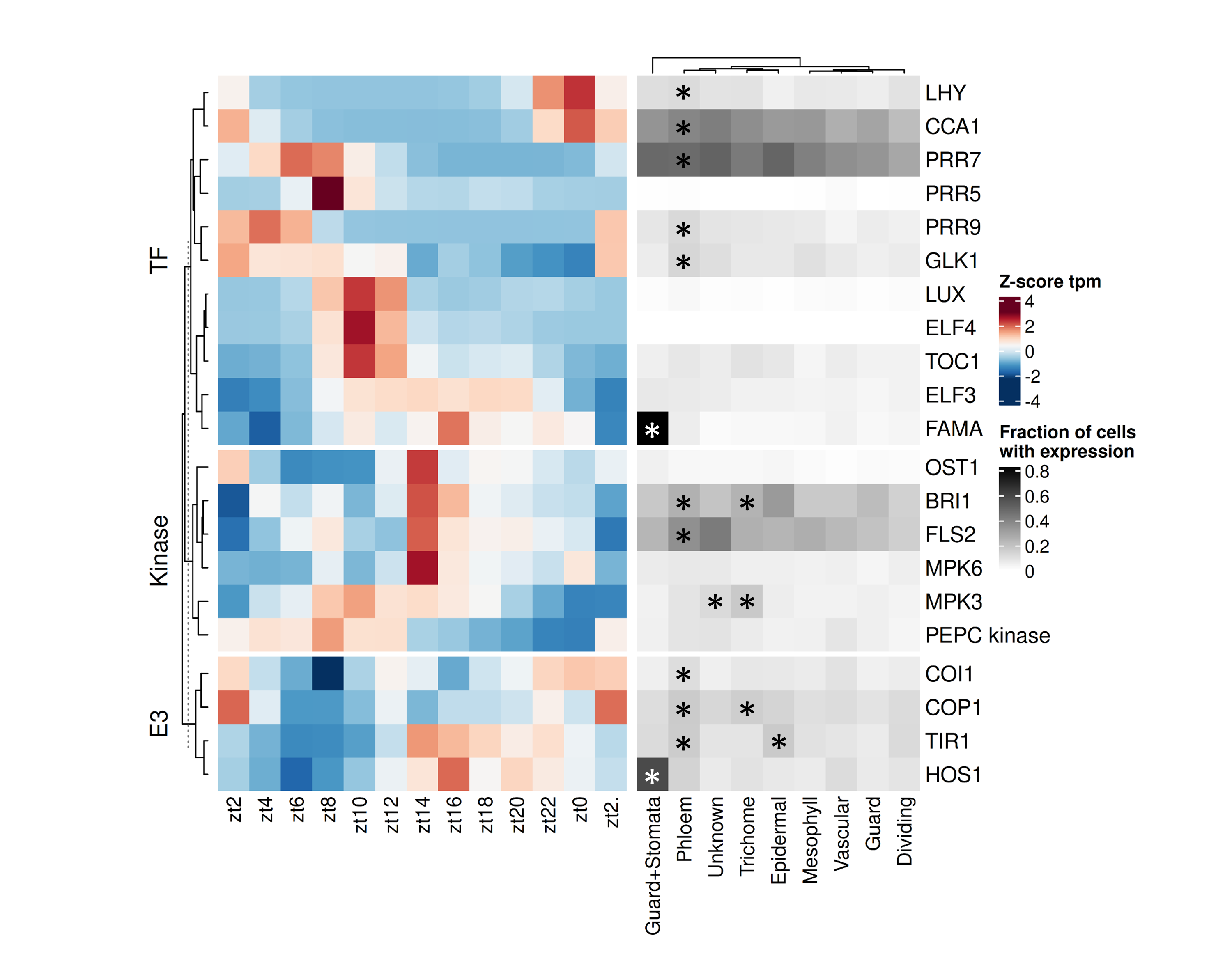

Supplement: Supplementary file 2 — Fig. S2. Heatmap of commonly known examples of kinases, E3 ligases and transcription factors. Heatmap showing expression (z‐score tpm) of all diurnal transcripts and fraction of cells with expression for each cell type. Stars indicate significantly differential expression in the cell type. Single‐cell data reanalysed from (Lee et al. 2025). [file PLB-28-1005-s002.tif]

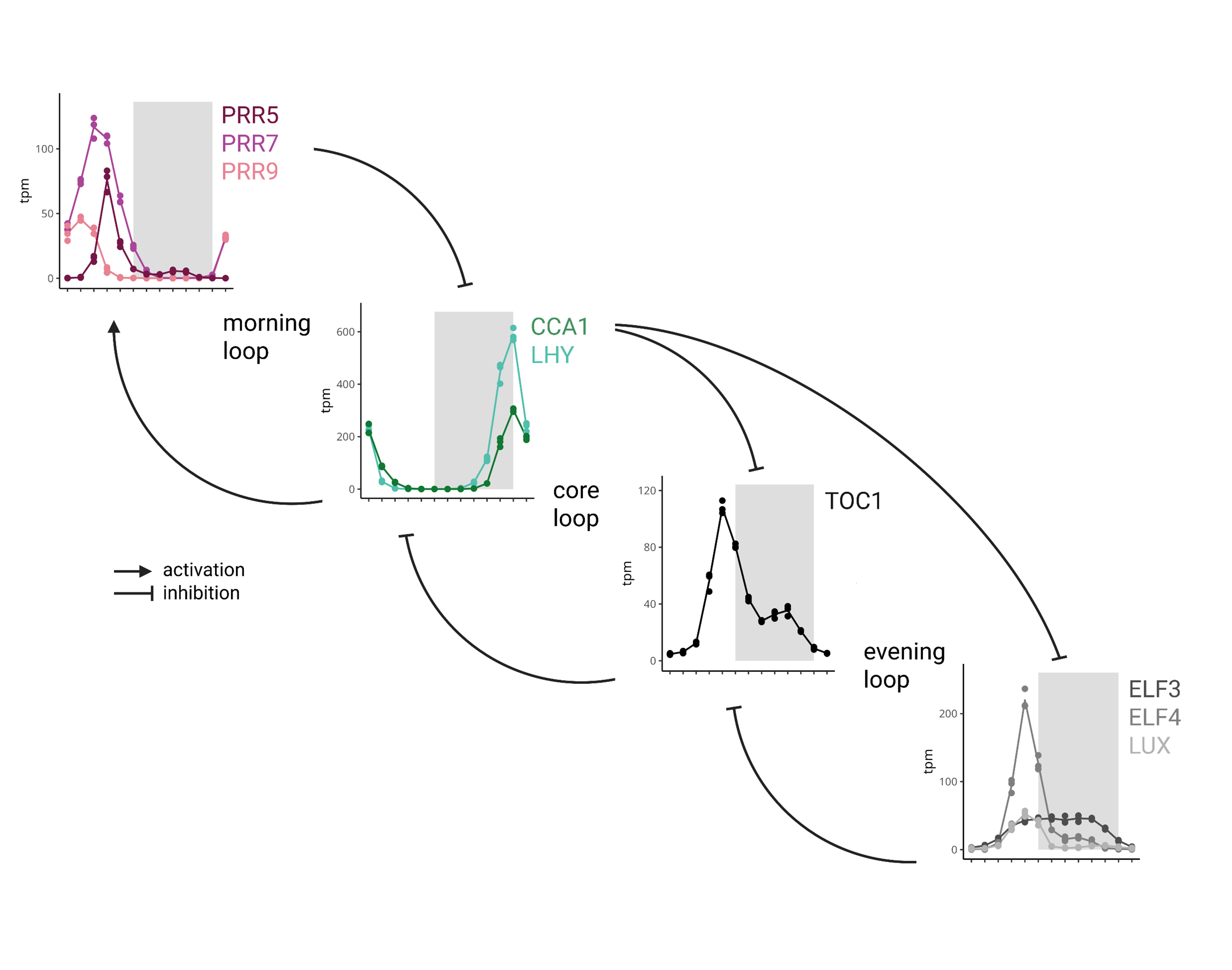

Supplement: Supplementary file 3 — Fig. S3. Expression patterns of core clock regulators. Transcripts per million (tpm) of all three replicates at each Zeitgeber timepoint. Mean values per timepoint are connected by a line. [file PLB-28-1005-s003.tif]
